# Preconception reproductive tract infections status and adverse pregnancy outcomes: a population-based retrospective cohort study

**DOI:** 10.1186/s12884-022-04836-3

**Published:** 2022-06-20

**Authors:** Mengyao Zeng, Liu Yang, Yanyan Mao, Yang He, Min Li, Jun Liu, Qianxi Zhu, Liang Chen, Weijin Zhou

**Affiliations:** 1 NHC Key Lab. of Reproduction Regulation, Shanghai Institute for Biomedical and Pharmaceutical Technologies, Fudan University, 400020 Shanghai, China; 2 NHC Key Laboratory of Birth Defects and Reproductive Health, Chongqing Population and Family Planning Science and Technology Research Institute, 200237 Chongqing, China

**Keywords:** Bacterial vaginosis, Candidiasis, Chlamydia, Preconception, Pregnancy outcomes, Reproductive tract infection, Syphilis

## Abstract

**Background:**

Reproductive tract infections can cause serious adverse outcomes for pregnant women such as spontaneous abortion and preterm birth. However, it is unclear whether maternal reproductive tract infection before pregnancy would also be related to any adverse pregnancy outcomes. This study aims to investigate the association of maternal preconception reproductive tract infections with subsequent adverse pregnancy outcomes.

**Methods:**

A retrospective cohort study was conducted in the Chongqing Municipality of China between April 2010 and December 2016. A total of 57,586 women (57,708 pregnancies) from all 39 counties of Chongqing who participated in the National Free Preconception Health Examination Project were included. They all took preconception examinations for gonorrhea, chlamydia, trichomoniasis, syphilis, bacterial vaginosis and candidiasis before pregnancy within one year. Primary outcomes included spontaneous abortion (< 28 weeks gestation), preterm birth (< 37 weeks gestation), macrosomia and low birthweight.

**Results:**

Of the 57,708 pregnancies, 2438 (4.22%) had at least one type of reproductive tract infections. Compared with women who were not infected with any reproductive tract infection before pregnancy, women with reproductive tract infections had a higher rate of spontaneous abortion (7.88% vs. 5.62%, p < 0.001). After analyzing by each infection, there were few significant associations between pre-pregnancy infections and adverse outcomes. Preconception syphilis infection was significantly associated with increased odds of spontaneous abortion (aOR = 2.07, 95%CI 1.50–2.85), induced abortion/labour due to medical reasons (aOR = 1.60, 95%CI 1.01–2.54) and preterm birth (aOR = 1.60, 95%CI 1.12–2.30) after adjusting for potential confounders. Preconception trichomoniasis was intended to relate to a higher risk of spontaneous abortion (aOR = 1.65, 95%CI 1.01–2.71), but its impact seemed to be attributed to its co-infection with other RTIs. Women who were chlamydia or bacterial vaginosis positive before pregnancy showed higher odds of macrosomia (aOR = 2.00, 95% CI 1.07–3.74 for chlamydia; aOR = 1.58, 95% CI 1.06–2.34 for bacterial vaginosis). Preconception bacterial vaginosis might also be associated with higher risks of very preterm birth (aOR = 2.16, 95%CI 1.23–3.78) and large for gestational age (aOR = 1.36, 95%CI 1.02–1.81).

**Conclusions:**

Women with infections of the genital tract before pregnancy might also have increased risks of subsequent adverse outcomes including spontaneous abortion, preterm birth and macrosomia.

**Supplementary Information:**

The online version contains supplementary material available at 10.1186/s12884-022-04836-3.

## Introduction

Reproductive tract infection (RTI) is the infection of the genital tract caused by viruses, bacteria, or other pathogens, which has become a major public health issue all over the world for its high and growing prevalence [1, 2]. According to the bulletin of World Health Organization, more than one million RTIs were acquired every day worldwide and the estimated global incidence for four types of RTI (chlamydia, gonorrhoea, trichomoniasis and syphilis) were 376.4 million in 2016 [3]. And in China, the national prevalence of RTIs reached 34.1% among the women of childbearing age in 2006 [4], which deserved our attention.

Some literature has addressed that RTI is harmful to pregnant women, which can cause serious health issues for both mothers and their foetuses, such as spontaneous abortion, stillbirth, congenital disease, and puerperal-sepsis [5–7]. However, few studies have focused on the maternal RTI status before pregnancy, so it is unclear whether maternal RTI during the preconception care is also related to any adverse pregnancy outcomes, or whether the impact of RTI on the foetus begins before the pregnancy through the intrauterine environment. Moreover, previous research mainly studied on one or two types of RTIs only, not taking the confounding from other infections or their mixed effects into consideration [8–10].

For this reason, we conducted this population-based retrospective cohort study to explore whether maternal preconception RTIs does have effects on any adverse outcomes, aiming to provide data support for improving decisions about both clinical practice and prevention work of RTIs during the preconception care. And since the biological mechanisms for RTI as a risk factor of adverse pregnancy outcomes are still not well understood, we also hope this study can come up with new issues for further research at the molecular level.

## Materials and methods

### Study population and design

**Fig. 1:**
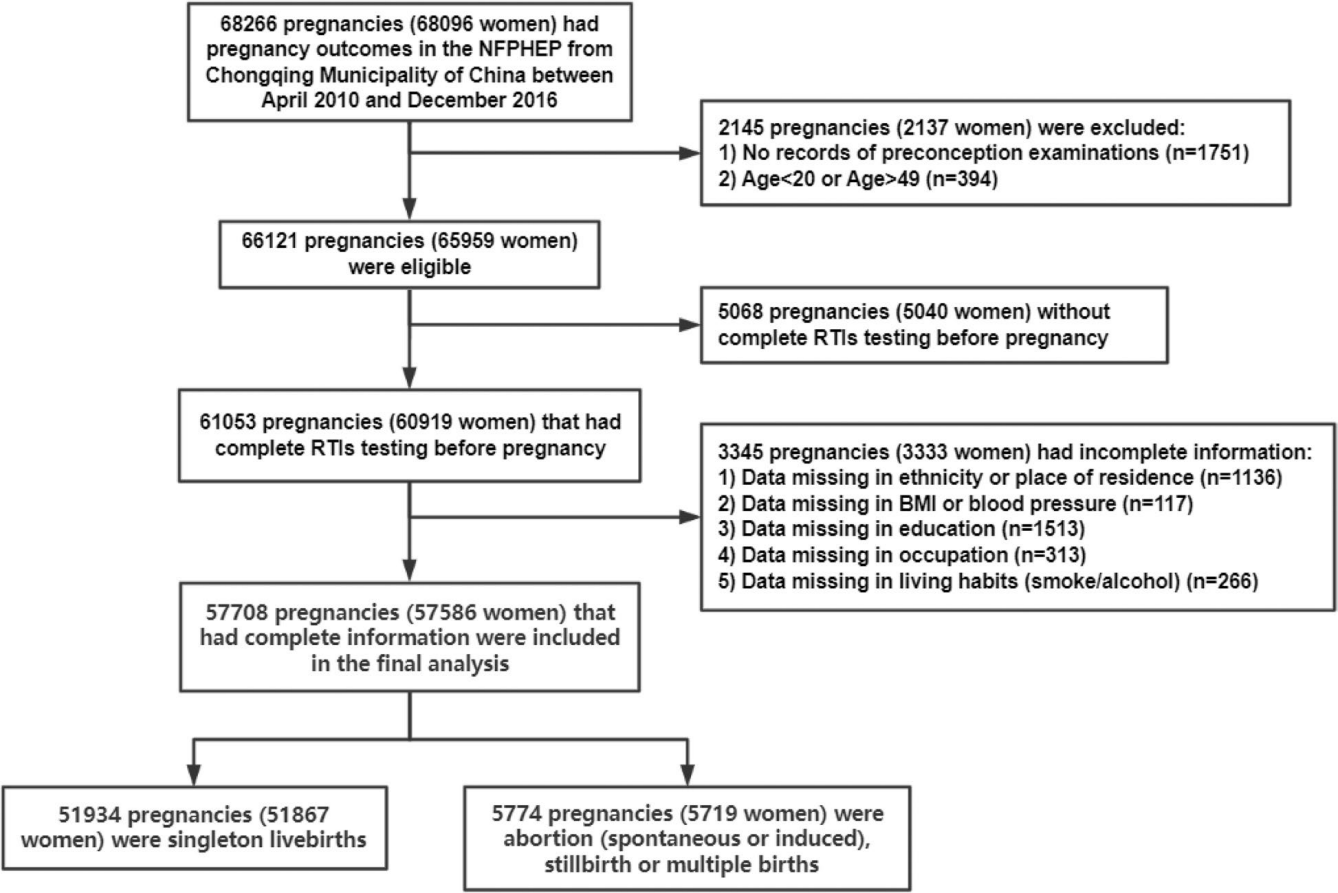
Flowchart for the study population

This retrospective cohort study was conducted in the Chongqing Municipality, west of China. The participants were women of childbearing age recruited from the National Free Preconception Health Examination Project (NFPHEP) between April 2010 and December 2016. NFPHEP was a national project launched to provide free preconception health examinations, consultations and risk assessments during their early pregnancy and postpartum follow-ups for couples who prepared to conceive, aiming to improve maternal and infant health in China. The detailed information about the procedures and implementation of this project has been well introduced previously [11, 12]. We collected data regarding the preconception care and following pregnancy outcomes of 68,096 women (68,266 pregnancies) women from the NFPHEP database of Chongqing. Based on the definition of childbearing women and the age requirement of Marriage Law in China, 65,959 women (66,121 pregnancies) between 20 and 49 years old with preconception examinations were eligible in this study. Women who failed to undergo complete RTIs testing during the preconception care were excluded. The flowchart of the study population was shown in Fig. 1. Our final analyses included 57,586 women (57,708 pregnancies) who had complete information on the basic characteristics that we were interested in, including ethnicity, educational level, occupation, place of residence, body mass index (BMI), blood pressure, lifestyle habits and history of childbearing. Of the included pregnancies, 51,934 had singleton livebirths.

The study had been approved by the Institutional Review Board of the Chinese Association of Maternal and Child Health Studies. Written informed consent was provided from all participants before enrollment.

### Procedures

When the participants enrolled in the NFPHEP, the baseline characteristics at preconception health examination, including demographic information (age, educational level, ethnicity, place of residence, and occupation), lifestyle habits (smoking and alcohol consumption), and childbearing history information (gravidity, parity, history of preterm, history of spontaneous abortion, and history of induced abortion) of participants were collected by the locally trained health workers with a standardized questionnaire. Blood samples, vaginal and cervical discharge of the participants were collected at the preconception health examinations as well. After enrolment, the participants were followed up by telephone calls every 3 months for a year to check for the status of their conception. If pregnant, their pregnancy outcomes, including abortion, stillbirth, neonate sex, gestational days, and birthweight, were collected from the medical records at the postpartum follow-up after delivery.

### Exposure measurement

In this study, we assessed six types of RTIs among the participants. Gonorrhea was identified based on colonial morphology and gram stain of the vaginal smear cultures that were incubated at 36ºC–37ºC in carbon dioxide for 24–48 h. The diagnosis of chlamydia infection was determined by golden colloid immunity diffuse test. Trichomoniasis was diagnosed by microscopic identification of typical motile trichomonas in a wet mount of vaginal discharge. The diagnosis of syphilis was made by the rapid plasma regain (RPR) test to screen for *Treponema pallidum* (TP) at first among the participants, and if the results were positive or equivocal, then treponema pallidum particle agglutination assay (TPPA) was used to detect antibodies specific for TP antigens to confirm. Bacterial vaginosis was identified by the Amsel criteria. A confirmed diagnosis of bacterial vaginosis should meet at least three of the four criteria: homogeneous white or yellow discharge; the presence of clue cells in vaginal discharge; pH of vaginal fluid > 4.5; Release of a fishy odor after adding 10% potassium hydroxide (KOH) solution on vaginal discharge [13]. The diagnosis of candidiasis infection was determined by the microscopic identification of fungal hyphae or yeast cells in a wet mount of vaginal discharge. These RTIs were all tested in the local laboratories affiliated to medical institutions under qualified quality control mechanisms. Choices regarding reagent kits used were decided by local county laboratories, all of which were approved by the China Food and Drug Administration [14]. Hypertension was defined as a systolic blood pressure ≥ 140 mmHg, and/or diastolic blood pressure ≥ 90 mmHg [15]. Maternal BMI was categorized into four groups according to the Working Group of China definition (< 18.5, 18.5–23.9, 24.0–27.9, or ≥ 28.0 kg/m^2^) [16, 17].

### Outcome definition

All pregnancy outcomes were identified according to the clinical diagnosis by the caring obstetrician. In this study, spontaneous abortion was defined according to clinical classification of obstetrics and gynecology in China as the loss of pregnancy before 28 weeks of gestation [12, 18, 19]. Induced abortion (or induced labour) due to medical reasons was defined as pregnancies that were terminated due to maternal serious diseases or abnormal conditions following the physicians’ advice. Abortions on self-request were excluded there. Stillbirth was defined as the death of a foetus at any time after the 28th week of pregnancy [20]. For outcomes in singleton livebirths, preterm birth was defined as delivery of a baby between 24 and 37 completed gestational weeks [21, 22], while very preterm birth was the delivery earlier than 32 completed gestational weeks [23]. Low birthweight (LBW) was defined as birthweight less than 2,500 g, while macrosomia was neonates with birth weight equal or above 4,000 g. Small for gestational age (SGA) or large for gestational age (LGA) was defined as birthweight < 10th or > 90th percentile, respectively, based on the Chinese neonatal birthweight curve for each gestational age established in 2015 [24].

### Statistical analysis

The proportions of maternal baseline characteristics and rates of adverse pregnancy outcomes according to the preconception RTI status were grouped and computed. Pearson Chi-square test or Fisher's exact test was used to examine the univariate associations of RTIs with categorical characteristics. Mean (SD) for neonatal continuous characteristics of the RTI-infected group and RTI-uninfected group were calculated and tested by one-way ANOVA analysis. Logistic regression models were used to explore the effect of each type of preconception RTIs on the risk of adverse birth outcomes. Results were presented as odds ratios (ORs) with their 95% confidence intervals (CIs) of the infected group compared to the women without any RTIs there. Adjusted odds ratios (aOR) with 95%CIs in the multivariate logistic regression models were calculated by adjusting for all potential confounders, including maternal age, maternal BMI, ethnicity, education level, occupation, place of residence, smoking, passive smoking, alcohol, hypertension, parity, history of preterm birth, history of spontaneous abortion and history of induced abortion at preconception health examination. For outcomes of women with singleton livebirths (preterm birth, very preterm birth, macrosomia, LGA, LBW, and SGA), aOR was additionally adjusted for neonate sex. Stratified analysis by infection status was used to assess whether the co-infections influenced the significant association between the independent infection and the adverse outcome. Subgroup analysis was used to examine the association between preconception RTIs and adverse pregnancy outcomes among different subgroups on the baseline characteristics. Statistical analyses were conducted using SAS statistical software (9.4 version). A 2-tailed level of 0.05 was considered the statistical significance.

## Results

Of the 57,708 pregnancies that had complete pre-pregnancy testing results of RTIs with pregnancy outcomes enrolled in our project, 2438 (4.22%) had at least one type of preconception RTIs (Table 1). Compared with women who were uninfected with any RTI, the infected women had a higher proportion of advanced maternal age (age 35 or more) (7.01% vs. 3.94%), overweight and obesity (14.56% vs. 11.81%), and were more likely to smoke (1.03% vs. 0.56%) or having hypertension (2.13% vs. 1.44%). As for the history of childbearing, women in the infected group had a higher percentage of multigravida (65.63% vs. 51.24%) and multipara (38.02% vs. 29.52%), with a clear higher proportion of having a spontaneous abortion (7.22% vs. 4.42%) or induced abortion (46.80% vs. 35.05%), *p* < 0.001 for all above comparisons in the history of childbearing.

**Table 1:** Maternal baseline characteristics with respect to preconception status of reproductive tract infection

Characteristics	RTI-infected	RTI-uninfected	*p*-value
No. of pregnancies (%)	2438 (4.22)	55,270 (95.78)	
Age, years			< 0.001
20–24	966 (39.62)	25,796 (46.67)	
25–29	993 (40.73)	21,849 (39.53)	
30–34	308 (12.63)	5449 (9.86)	
35–49	171 (7.01)	2176 (3.94)	
BMI, kg/m^2^			< 0.001
< 18.5	326 (13.37)	8292 (15.01)	
18.5–23.9	1757 (72.07)	40,448 (73.18)	
24–27.9	308 (12.63)	5579 (10.09)	
≥ 28	47 (1.93)	951 (1.72)	
Ethnicity			0.055
Han	2307 (94.63)	52,759 (95.46)	
Others	131 (5.37)	2511 (4.54)	
Education			< 0.001
Primary or below	160 (6.56)	2368 (4.28)	
Middle school	1096 (44.95)	24,532 (44.39)	
High school	536 (21.99)	13,758 (24.89)	
College or above	646 (26.50)	14,612 (26.44)	
Place of residence			0.005
Non-agricultural	699 (28.67)	14,433 (26.11)	
Agricultural	1739 (71.33)	40,837 (73.89)	
Occupation			< 0.001
Peasant	1004 (41.18)	24,768 (44.81)	
labor worker	249 (10.21)	5670 (10.26)	
Merchant	253 (10.38)	5975 (10.81)	
service staff	82 (3.36)	1957 (3.54)	
Housewife	184 (7.55)	3308 (5.99)	
Civil servant	414 (16.98)	8448 (15.28)	
Others	252 (10.34)	5144 (9.31)	
Smoking			0.004
No	2413 (98.97)	54,958 (99.44)	
Yes	25 (1.03)	312 (0.56)	
Passive smoking			0.582
No	2060 (84.50)	46,926 (84.90)	
Yes	378 (15.50)	8344 (15.10)	
Alcohol			0.339
No	2295 (94.13)	52,276 (94.58)	
Yes	143 (5.87)	2994 (5.42)	
Gravidity			< 0.001
0	838 (34.37)	26,948 (48.76)	
≥ 1	1600 (65.63)	28,322 (51.24)	
Parity			< 0.001
0	1511 (61.98)	38,957 (70.48)	
≥ 1	927 (38.02)	16,313 (29.52)	
History of preterm birth			0.945
No	2433 (99.79)	55,153 (99.79)	
Yes	5 (0.21)	117 (0.21)	
History of spontaneous abortion			< 0.001
No	2262 (92.78)	52,829 (95.58)	
Yes	176 (7.22)	2441 (4.42)	
History of induced abortion			< 0.001
No	1297 (53.20)	35,900 (64.95)	
Yes	1141 (46.80)	19,370 (35.05)	
Hypertension			0.006
No	2386 (97.87)	54,473 (98.56)	
Yes	52 (2.13)	797 (1.44)	
Interval			< 0.001
0–3 months	1572 (64.74)	39,591 (72.02)	
4–6 months	526 (21.66)	9653 (17.56)	
7–12 months	330 (13.59)	5727 (10.42)	
Missing	10	299	

Interval: the period between the date of RTIs testing and the date of the last menstrual period of pregnant women

*BMI* body mass index (calculated as the weight in kilograms divided by height in meters squared)

In relation to the pregnancy outcomes, women with preconception RTIs had a higher rate of almost all adverse outcomes including spontaneous abortion, preterm birth, macrosomia and LBW compared to the uninfected women, although no significant differences were observed in most outcomes except spontaneous abortion (7.88% vs. 5.62%, *p* < 0.001) (Table 2).

**Table 2:** Associations of preconception RTI status with pregnancy outcomes

Pregnancy outcomes	RTI-infected	RTI-uninfected	*p*-value
Categorical variables	n /N (%)	n /N (%)	
Spontaneous abortion	192 /2438 (7.88)	3105 /55270 (5.62)	** < 0.001**
Induced abortion / labor	92 /2438 (3.77)	1758 /55270 (3.18)	0.104
Stillbirth	2 /2438 (0.08)	121 /55270 (0.22)	0.152
Neonate sex (Male)	1104 /2119 (52.10)	25,809 /49599 (52.04)	0.953
Preterm Birth	177 /2124 (8.33)	3874 /49763 (7.78)	0.356
Very Preterm Birth	41 /2124 (1.93)	731 /49763 (1.47)	0.085
Macrosomia	105 /2110 (4.98)	2061 /49292 (4.18)	0.075
LGA	232 /2108 (11.01)	4967 /49224 (10.09)	0.173
LBW	31 /2110 (1.47)	548 /49292 (1.11)	0.128
SGA	137 /2108 (6.50)	2848 /49224 (5.79)	0.171
Continuous variables	Mean ± SD (N)	Mean ± SD (N)	
Gestational age, days	274.86 ± 15.07 (2124)	274.97 ± 14.58 (49,810)	0.730
Neonate birthweight, g	3303.44 ± 413.10 (2110)	3299.99 ± 390.46 (49,299)	0.691

Abbreviation: *LGA*, large for gestational age (birthweight > 90th percentile), *LBW* low birthweight (birthweight < 2,500 g), *SGA* small for gestational age (birthweight < 10th)

The associations of each type of RTI with adverse pregnancy outcomes were presented in Table 3. Among all comparisons between each preconception RTI and adverse outcomes, only four RTIs showed a significant negative impact on birth outcomes, including syphilis, trichomoniasis, chlamydia and bacterial vaginosis. Further analysis of these significant associations stratified by independent RTI or co-infections was shown in Table 4. After adjusting for potential confounders, preconception syphilis infection was associated with an increased risk of spontaneous abortion (aOR = 2.07, 95%CI 1.50–2.85), regardless of an independent infection or co-infection with other RTIs. Preconception syphilis infection was also associated with higher risks of induced abortion/labour due to medical reasons (aOR = 1.60, 95%CI 1.01–2.54) and preterm birth (aOR = 1.60, 95%CI 1.12–2.30), especially in the infection with syphilis alone. Preconception trichomoniasis was intended to relate to a higher risk of spontaneous abortion (aOR = 1.65, 95%CI 1.01–2.71), but the infection alone seemed to be no longer significantly associated with spontaneous abortion (aOR = 1.27, 95%CI 0.68–2.38). Compared with women without any RTI during the preconception care, those who were chlamydia or bacterial vaginosis positive both had higher odds of macrosomia (aOR = 2.00, 95% CI 1.07–3.74 for chlamydia; aOR = 1.58, 95% CI 1.06–2.34 for bacterial vaginosis), and the possible harmful effects were mainly from the separate infection. Preconception bacterial vaginosis might also be associated with higher risks of very preterm birth (aOR = 2.16, 95%CI 1.23–3.78) and LGA (aOR = 1.36, 95%CI 1.02–1.81), though the associations seemed no longer significantly in women with bacterial vaginosis alone. In subgroup analyses stratified by maternal characteristics, these associations did not markedly change in overall, although some subgroups no longer presented a statistically significant risk (Supplementary Figure S1-S2).

**Table 3:** Associations of six types of RTIs during preconception care with adverse pregnancy outcomes

Type of infection	Trichomoniasis	Syphilis	Gonorrhea	Chlamydia	Bacterial vaginosis	Candidiasis	RTIs-uninfected
Among 57,708 pregnancies that had pregnancy outcomes							
**Spontaneous abortion**							
n /N (%)	18 /184 (9.78)	45 /348 (12.93)	2 /79 (2.53)	11 /138 (7.97)	40 /477 (8.39)	90 /1297 (6.94)	3105 /55270 (5.62)
Crude OR (95%CI)	1.82 (1.12–2.97)	2.50 (1.82–3.42)	0.44 (0.11–1.78)	1.46 (0.79–2.70)	1.54 (1.11–2.13)	1.25 (1.01–1.56)	1.00 (Ref.)
Adjusted OR^a^ (95%CI)	**1.65 (1.01–2.71)**	**2.07 (1.50–2.85)**	0.38 (0.09–1.56)	1.36 (0.73–2.55)	1.33 (0.96–1.85)	1.14 (0.92–1.42)	1.00 (Ref.)
**Induced abortion / labour due to medical reasons**							
n /N (%)	11 /184 (5.98)	20 /348 (5.75)	0 /79 (0)	3 /138 (2.17)	21 /477 (4.40)	39 /1297 (3.01)	1758 /55270 (3.18)
Crude OR (95%CI)	1.94 (1.05–3.57)	1.86 (1.18–2.92)	/	0.68 (0.22–2.13)	1.40 (0.90–2.18)	0.94 (0.68–1.30)	1.00 (Ref.)
Adjusted OR^a^ (95%CI)	1.75 (0.95–3.26)	**1.60 (1.01–2.54)**	/	0.65 (0.21–2.05)	1.18 (0.76–1.84)	0.84 (0.61–1.17)	1.00 (Ref.)
**Stillbirth**							
n /N (%)	0 /184 (0)	0 /348 (0)	0 /79 (0)	0 /138 (0)	1 /477 (0.21)	1 /1297 (0.08)	121 /55270 (0.22)
Crude OR (95%CI)	/	/	/	/	0.96 (0.13–6.87)	0.35 (0.05–2.52)	1.00 (Ref.)
Adjusted OR^a^ (95%CI)	/	/	/	/	0.89 (0.12–6.41)	0.35 (0.05–2.52)	1.00 (Ref.)
Among 51,934 pregnancies with singleton livebirths							
**Preterm Birth**							
n /N (%)	9 /152 (5.92)	34 /276 (12.32)	3 /76 (3.95)	11 /125 (8.80)	34 /407 (8.35)	91 /1156 (7.87)	3874 /49763 (7.78)
Crude OR (95%CI)	0.75 (0.38–1.46)	1.66 (1.16–2.39)	0.49 (0.15–1.55)	1.14 (0.62–2.12)	1.08 (0.76–1.54)	1.01 (0.82–1.26)	1.00 (Ref.)
Adjusted OR^b^ (95%CI)	0.73 (0.37–1.44)	**1.60 (1.12–2.30)**	0.48 (0.15–1.52)	1.14 (0.61–2.13)	1.08 (0.76–1.54)	1.01 (0.81–1.25)	1.00 (Ref.)
**Very Preterm Birth**							
n /N (%)	2 /152 (1.32)	7 /276 (2.54)	0 /76 (0)	3 /125 (2.40)	13 /407 (3.19)	19 /1156 (1.64)	731 /49763 (1.47)
Crude OR (95%CI)	0.89 (0.22–3.62)	1.75 (0.82–3.71)	/	1.65 (0.52–5.20)	2.22 (1.27–3.87)	1.12 (0.71–1.77)	1.00 (Ref.)
Adjusted OR^b^ (95%CI)	0.89 (0.22–3.60)	1.64 (0.77–3.51)	/	1.79 (0.57–5.67)	**2.16 (1.23–3.78)**	1.15 (0.72–1.82)	1.00 (Ref.)
**Macrosomia**							
n /N (%)	5 /152 (3.29)	9 /273 (3.30)	1 /75 (1.33)	11 /125 (8.80)	27 /405 (6.67)	55 /1148 (4.79)	2061 /49292 (4.18)
Crude OR (95%CI)	0.78 (0.32–1.90)	0.78 (0.40–1.52)	0.31 (0.04–2.23)	2.21 (1.19–4.12)	1.64 (1.11–2.43)	1.15 (0.88–1.52)	1.00 (Ref.)
Adjusted OR^b^ (95%CI)	0.69 (0.28–1.69)	0.76 (0.39–1.48)	0.32 (0.04–2.29)	**2.00 (1.07–3.74)**	**1.58 (1.06–2.34)**	1.06 (0.81–1.40)	1.00 (Ref.)
**LGA**							
n /N (%)	13 /152 (8.55)	32 /273 (11.72)	5 /75 (6.67)	18 /125 (14.40)	55 /405 (13.58)	115 /1146 (10.03)	4967 /49224 (10.09)
Crude OR (95%CI)	0.83 (0.47–1.47)	1.18 (0.82–1.71)	0.64 (0.26–1.58)	1.50 (0.91–2.48)	1.40 (1.05–1.87)	0.99 (0.82–1.21)	1.00 (Ref.)
Adjusted OR^b^ (95%CI)	0.79 (0.45–1.40)	1.13 (0.78–1.64)	0.64 (0.26–1.59)	1.41 (0.85–2.32)	**1.36 (1.02–1.81)**	0.96 (0.79–1.17)	1.00 (Ref.)
**LBW**							
n /N (%)	1 /152 (0.66)	6 /273 (2.20)	1 /75 (1.33)	2 /125 (1.60)	5 /405 (1.23)	17 /1148 (1.48)	548 /49292 (1.11)
Crude OR (95%CI)	0.59 (0.08–4.22)	2.00 (0.89–4.51)	1.20 (0.17–8.66)	1.45 (0.36–5.87)	1.11 (0.46–2.70)	1.34 (0.82–2.18)	1.00 (Ref.)
Adjusted OR^b^ (95%CI)	0.60 (0.08–4.28)	1.85 (0.82–4.20)	1.14 (0.16–8.28)	1.39 (0.34–5.64)	1.13 (0.46–2.73)	1.31 (0.80–2.14)	1.00 (Ref.)
**SGA**							
n /N (%)	8 /152 (5.26)	15 /273 (5.49)	0 /75 (0)	9 /125 (7.20)	30 /405 (7.41)	78 /1146 (6.81)	2848 /49224 (5.79)
Crude OR (95%CI)	0.91 (0.44–1.85)	0.95 (0.56–1.60)	/	1.26 (0.64–2.49)	1.30 (0.90–1.89)	1.19 (0.94–1.50)	1.00 (Ref.)
Adjusted OR^b^ (95%CI)	0.93 (0.45–1.89)	0.98 (0.58–1.65)	/	1.27 (0.64–2.51)	1.32 (0.91–1.93)	1.19 (0.94–1.50)	1.00 (Ref.)

*OR* odds ratio

^a^Adjusted OR were adjusted for maternal age, maternal preconception BMI, ethnicity, education level, occupation, place of residence, smoking, passive smoking, alcohol, parity, history of preterm birth, history of spontaneous abortion, history of induced abortion and hypertension

^b^Adjusted OR were additionally adjusted for neonate sex

**Table 4:** Selected associations of preconception RTIs with adverse pregnancy outcomes, stratifying by infection status

	Infected	RTI-uninfected	Crude OR (95%CI)	Adjusted OR (95%CI)
	n /N (%)	n /N (%)		
**Spontaneous abortion**^a^				
Trichomoniasis (Independent)	11 /142 (7.75)	3105 /55270 (5.62)	1.41 (0.76–2.62)	1.27 (0.68–2.38)
Trichomoniasis (Co-infection)	7 /42 (16.67)	3105 /55270 (5.62)	3.36 (1.49–7.57)	**3.08 (1.34–7.07)**
Syphilis (Independent)	38 /306 (12.42)	3105 /55270 (5.62)	2.38 (1.70–3.36)	**1.95 (1.37–2.76)**
Syphilis (Co-infection)	7 /42 (16.67)	3105 /55270 (5.62)	3.36 (1.49–7.57)	**3.11 (1.35–7.18)**
**Induced abortion / labour due to medical reasons**^a^				
Syphilis (Independent)	20 /306 (6.54)	1758 /55270 (3.18)	2.13 (1.35–3.36)	**1.83 (1.15–2.91)**
Syphilis (Co-infection)	0 /42 (0)	1758 /55270 (3.18)	/	/
**Preterm Birth**^b^				
Syphilis (Independent)	30 /242 (12.40)	3874 /49763 (7.78)	1.68 (1.14–2.46)	**1.60 (1.09–2.35)**
Syphilis (Co-infection)	4 /34 (11.76)	3874 /49763 (7.78)	1.59 (0.56–4.50)	1.61 (0.56–4.57)
**Very Preterm Birth**^b^				
Bacterial vaginosis (Independent)	10 /367 (2.72)	731 /49763 (1.47)	1.88 (1.00–3.54)	1.81 (0.96–3.42)
Bacterial vaginosis (Co-infection)	3 /40 (7.50)	731 /49763 (1.47)	5.44 (1.67–17.68)	**5.73 (1.75–18.77)**
**Macrosomia**^b^				
Chlamydia (Independent)	11 /99 (11.11)	2061 /49292 (4.18)	2.87 (1.53–5.37)	**2.59 (1.38–4.87)**
Chlamydia (Co-infection)	0 /26 (0)	2061 /49292 (4.18)	/	/
Bacterial vaginosis (Independent)	24 /365 (6.58)	2061 /49292 (4.18)	1.61 (1.06–2.45)	**1.54 (1.01–2.34)**
Bacterial vaginosis (Co-infection)	3 /40 (7.50)	2061 /49292 (4.18)	1.86 (0.57–6.03)	1.75 (0.54–5.72)
**LGA**^**2**^				
Bacterial vaginosis (Independent)	49 /365 (13.42)	4967 /49224 (10.09)	1.38 (1.02–1.87)	1.34 (0.99–1.81)
Bacterial vaginosis (Co-infection)	6 /40 (15.00)	4967 /49224 (10.09)	1.58 (0.66–3.75)	1.55 (0.65–3.72)

Trichomoniasis (Co-infection): mainly combined with Bacterial vaginosis (45%), Candidiasis (19%) and Syphilis (17%)

Syphilis (Co-infection): mainly combined with Bacterial vaginosis (50%), Trichomoniasis (22%) and Gonorrhea (22%)

Chlamydia (Co-infection): mainly combined with Bacterial vaginosis (53%), Trichomoniasis (21%) and Gonorrhea (18%)

Bacterial vaginosis (Co-infection): mainly combined with Candidiasis (46%), Trichomoniasis (17%) and Gonorrhea (17%)

^a^Adjusted OR were adjusted for maternal age, maternal preconception BMI, ethnicity, education level, occupation, place of residence, smoking, passive smoking, alcohol, parity, history of preterm birth, history of spontaneous abortion, history of induced abortion and hypertension

^b^Adjusted OR were additionally adjusted for neonate sex

## Discussion

In our study, we explored the associations between maternal preconception RTIs and adverse pregnancy outcomes. We found that preconception syphilis alone was associated with increased risks of spontaneous abortion, induced abortion/labour due to medical reasons and preterm birth. Both pre-pregnancy chlamydia and bacterial vaginosis infection alone seemed to be significantly associated with a higher risk of macrosomia. Moreover, maternal pre-pregnancy trichomoniasis infection was intended to relate to a higher risk of spontaneous abortion, and bacterial vaginosis infection was associated with very preterm birth, though the associations might be influenced by the co-infections with other RTIs.

To our knowledge, the association of maternal infections (especially related to the genital tract or urinary tract) during pregnancy with perinatal outcomes had been well studied. Most positive findings were consistent, that was, maternal infections during pregnancy, regardless of at early pregnancy or late pregnancy, were associated with increased risks of preterm birth and LBW [7, 25]. A few studies reported that the infections were also associated with neonatal pneumonia or perinatal mortality [26]. Some discoveries in mechanism research can explain part of these findings. Bacterial infection, such as bacterial vaginosis, would trigger the anti-inflammatory and mucosal homeostatic responses of pregnant women to the infection, which might determine the outcomes of pregnancy [27]. As for viral infection, the possible mechanisms included production of toxins metabolic byproducts, which might induce uterine contractions or placental infection, leading to abortion or stillbirth [28]. Our findings suggested that women with some certain RTIs before their pregnancies might also increase their risks of a variety of adverse outcomes, implying that RTIs prevention, screening and treatment for women preparing to pregnant should be strengthened and prioritized to reduce the risk of adverse pregnancy outcomes, and that monitoring the risk of spontaneous abortion, preterm birth, and macrosomia in infected pregnant women should not be ignored.

As for the outcome of spontaneous abortion and preterm birth, our finding was consistent with previous studies of infections during pregnancy [29–31]. Among the RTIs, preconception syphilis infection was significantly associated with spontaneous abortion and preterm birth with odds ratios of 2.07 and 1.60 between the infected and uninfected group after adjusting potential confounders. For trichomoniasis, its possible impact on spontaneous abortion has not been reported before, while a systematic review found that antenatal trichomoniasis was associated with increased risk of preterm birth, premature rupture of membranes (PROM) and SGA [32]. Since its impact on spontaneous abortion seemed to be contributed to co-infection with other RTI in this study (mainly combined with bacterial vaginosis, candidiasis, and syphilis), more evidence might be needed for the positive association between trichomoniasis infection and spontaneous abortion. In addition, we found that women who acquire chlamydia and bacterial vaginosis infections before pregnancy were at increased risk of delivering macrosomia infants. It seemed to be an unexplainable finding that no other studies had similar results, regardless of before or during the pregnancy. Such impact on macrosomia in this study might be influenced by other risk factors we had no information such as gestational diabetes status during pregnancy.

One possible explanation for our findings is that pre-pregnancy RTIs might interfere with follicular growth, oocyte development, and maturation, resulting in poor embryo quality and subsequent adverse outcomes [33, 34]. The issue that how long it takes to eliminate or lessen the effects of these infections on adverse outcomes is needed to study for women with some RTIs but want to have a baby. Another possibility is that women who experience RTIs prior to pregnancy might be more likely to have RTIs during pregnancy [35, 36], which would be associated with increased risks of adverse outcomes.

Since all RTIs testing was conducted within one year before pregnancy in this study, and subgroup analysis showed that the associations between preconception RTI and adverse outcomes were still significantly among women with the longest interval between the RTIs testing and the pregnancy (Supplementary Figure S1-S2), further studies are needed to investigate the length of recommended intervals for women with different types of RTI between testing or cure and pregnancy.

This is one of the very few studies exploring the impact of preconception RTI status on pregnancy outcomes. The strength of our study is the large cohort based on an unselected population. For this cohort, we recruited more than 60,000 participants covering all 39 counties of Chongqing Municipality in China, with strict quality controls on exposure measurement and information collection. However, our study also had some limitations. Firstly, the NFPHEP did not test the RTI status of participants again before or during pregnancy, therefore, whether the infected women during preconception care had been cured before their pregnancies were unclear. Although the lack of information created some uncertainty, we believed that the significant risk of adverse outcomes in women with preconception RTIs might be underestimated, because women with infection would receive corresponding treatments according to the official guideline and obstetricians of NFPHEP. Second, the information on pregnancy complications such as gestational diabetes mellitus and gestational hypertension were not collected in the database, so we could not adjust them in the multivariable analysis, which might influence our findings. Third, our participants were all pregnant women, so the analysis for the impact of RTIs on infertility was not available, though fecundity should be an important outcome.

## Conclusion

Overall, in this large retrospective cohort study conducted in the southwest of China, preconception RTIs, such as syphilis and bacterial vaginosis, were associated with increased risks of many adverse pregnancy outcomes including spontaneous abortion, preterm birth and macrosomia. Although some immunological researches can explain part of similar findings during pregnancy, the mechanisms by which infection before pregnancy also has impacts on adverse outcomes is still unclear. Further high-quality prospective studies, which include information on RTI status and other complications of mothers during pregnancy, are needed to compare the effect of RTIs before and during pregnancy on adverse outcomes. Given the relatively limited health resources and heavy burden of RTIs in China, the prevention and early detection of RTIs for women preparing to pregnant, and the appropriate treatment and management for women with RTIs before pregnancy and during pregnancy might be helpful to reduce the risks of adverse pregnancy outcomes.

## Supplementary Information

**Additional file 1: Figure S1.** Subgroup analysis of spontaneous abortion in the women with preconception syphilis infection compared to the RTI-uninfected women. **Figure S2.** Subgroup analysis of preterm birth in the women with preconception syphilis infection compared to the RTI-uninfected women.

## Data Availability

All data relevant to the study are included in the article or uploaded as supplementary information.
