# Supplementary material for: Preconception reproductive tract infections status and adverse pregnancy outcomes: a population-based retrospective cohort study
Source: BMC Pregnancy Childbirth. 2022 Jun 20;22:501. doi: 10.1186/s12884-022-04836-3 (PMC9208112; doi:10.1186/s12884-022-04836-3)
Supplement: Supplementary file 1 — Additional file 1: Figure S1. Subgroup analysis of spontaneous abortion in the women with preconception syphilis infection compared to the RTI-uninfected women. Figure S2. Subgroup analysis of preterm birth in the women with preconception syphilis infection compared to the RTI-uninfected women. [file 12884_2022_4836_MOESM1_ESM.pdf]

Figure S1 Subgroup analysis of spontaneous abortion in the women with preconception syphilis infection compared to the RTI-uninfected women

	N (Syphilis)	N (RTI-uninfected)	aOR[1] (95%CI)
**Age, years**			
20-24	134	25796	1.76 (0.92-3.38)
25-29	115	21849	2.23 (1.26-3.93)
30-34	58	5449	1.43 (0.64-3.20)
35-49	41	2176	2.86 (1.46-5.64)
**BMI, kg/m^2**			
<18.5	37	8292	1.47 (0.44-4.89)
18.5-23.9	242	40448	2.10 (1.42-3.11)
24-27.9	56	5579	2.31 (1.11-4.79)
≥28	13	951	1.53 (0.31-7.55)
**Education**			
Primary or below	48	2368	2.22 (1.02-4.81)
Middle school	201	24532	2.33 (1.55-3.52)
High school	63	13758	1.78 (0.76-4.17)
College or above	36	14612	0.46 (0.06-3.41)
**Ethnicity**			
Han	327	52759	2.11 (1.52-2.94)
Others	21	2511	1.55 (0.34-7.09)
**Place of residence**			
Non-agricultural	61	14433	1.13 (0.44-2.88)
Agricultural	287	40837	2.28 (1.61-3.22)
**Passive smoking**			
No	301	46926	2.13 (1.51-3.00)
Yes	47	8344	1.77 (0.67-4.68)
**Alcohol**			
No	324	52276	2.05 (1.46-2.86)
Yes	24	2994	2.66 (0.76-9.32)
**Gravidity**			
0	99	26948	1.29 (0.52-3.20)
⩾1	249	28322	2.21 (1.55-3.13)
**Parity**			
0	207	38957	2.32 (1.47-3.68)
⩾1	141	16313	1.83 (1.17-2.88)
**History of spontaneous abortion**			
No	321	52829	2.06 (1.47-2.89)
Yes	27	2441	1.99 (0.64-6.18)
**History of induced abortion**			
No	188	35900	1.60 (0.97-2.64)
Yes	160	19370	2.58 (1.68-3.96)
**Interval**			
0-3 month	246	39591	1.93 (1.28-2.91)
4-6 month	56	9653	1.72 (0.77-3.86)
7-12 month	44	5727	3.07 (1.50-6.27)

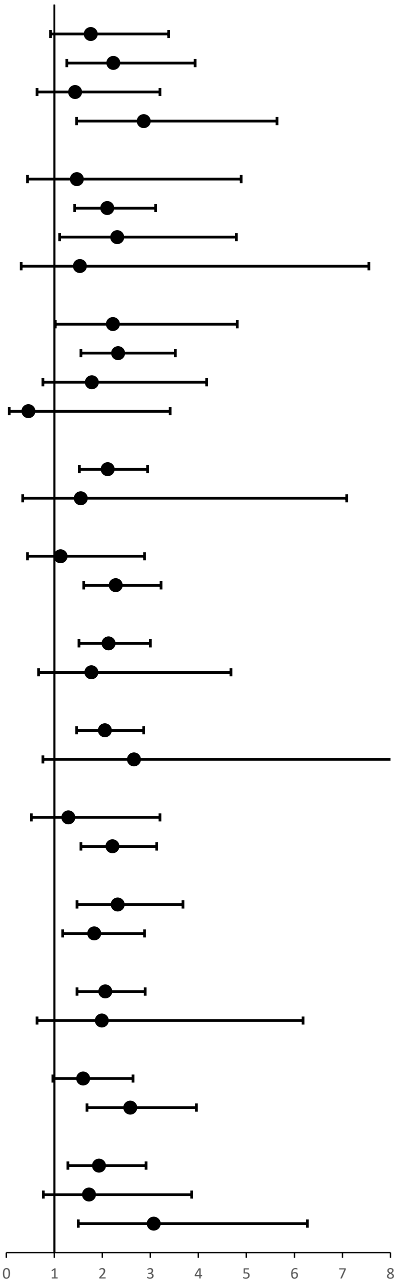

[1]aOR was adjusted for maternal age, maternal pregestational BMI, ethnicity, education level, occupation, place of residence, smoking, passive smoking, alcohol, parity, history of preterm birth, history of spontaneous abortion, history of induced abortion and hypertension.

Interval: the period between the date of RTIs testing and the date of the last menstrual period of pregnant women

Figure S2 Subgroup analysis of preterm birth in the women with preconception syphilis infection compared to the RTI-uninfected women

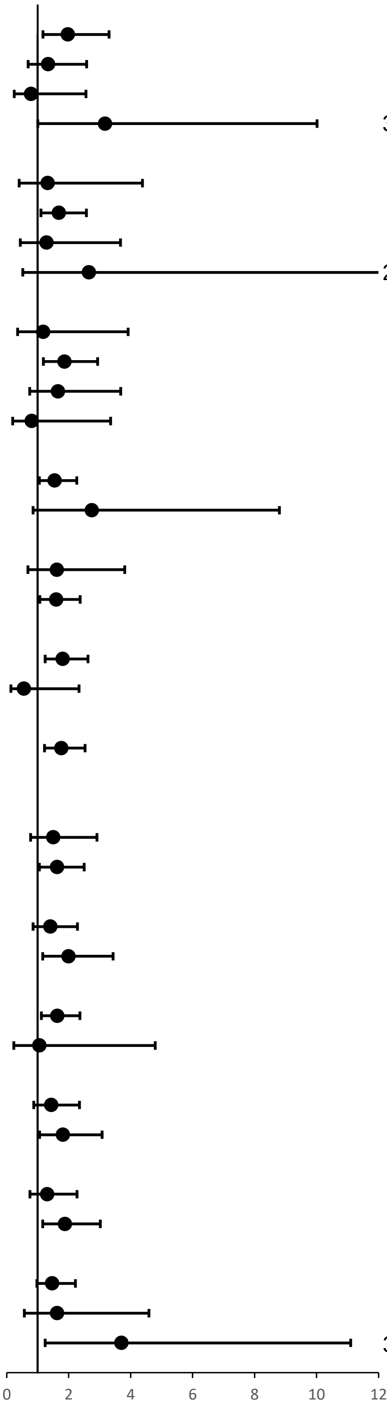

	N (Syphilis)	N (RTI-uninfected)	aOR[1] (95%CI)
**Age, years**			
20-24	119	23935	1.97 (1.17-3.30)
25-29	93	19598	1.33 (0.69-2.58)
30-34	43	4651	0.78 (0.24-2.56)
35-49	21	1626	3.17 (1.00-10.02)
**BMI, kg/m^2**			
<18.5	31	7585	1.32 (0.40-4.38)
18.5-23.9	198	36595	1.68 (1.10-2.57)
24-27.9	37	4828	1.28 (0.44-3.67)
≥28	10	802	2.65 (0.52-13.56)
**Education**			
Primary or below	31	2057	1.17 (0.35-3.92)
Middle school	157	22015	1.86 (1.18-2.93)
High school	54	12570	1.65 (0.74-3.68)
College or above	34	13168	0.80 (0.19-3.35)
**Ethnicity**			
Han	259	47548	1.54 (1.05-2.26)
Others	17	2262	2.74 (0.85-8.80)
**Place of residence**			
Non-agricultural	48	12873	1.61 (0.68-3.81)
Agricultural	228	36937	1.59 (1.07-2.37)
**Passive smoking**			
No	237	42211	1.80 (1.24-2.62)
Yes	39	7599	0.55 (0.13-2.33)
**Alcohol**			
No	256	47069	1.76 (1.22-2.53)
Yes	20	2741	/
**Gravidity**			
0	91	25023	1.50 (0.77-2.91)
⩾1	185	24787	1.62 (1.05-2.50)
**Parity**			
0	176	35985	1.40 (0.85-2.28)
⩾1	100	13825	1.99 (1.16-3.43)
**History of spontaneous abortion**			
No	256	47612	1.63 (1.12-2.36)
Yes	20	2198	1.05 (0.23-4.79)
**History of induced abortion**			
No	159	32779	1.43 (0.87-2.35)
Yes	117	17031	1.81 (1.06-3.08)
**Neonate sex**			
Male	134	25809	1.30 (0.75-2.27)
Female	140	23790	1.87 (1.16-3.02)
**Interval**			
0-3 month	207	36318	1.46 (0.97-2.22)
4-6 month	43	8377	1.62 (0.57-4.59)
7-12 month	26	4860	3.70 (1.24-11.10)

[1]aOR was adjusted for maternal age, maternal pregestational BMI, ethnicity, education level, occupation, place of residence, smoking, passive smoking, alcohol, parity, history of preterm birth, history of spontaneous abortion, history of induced abortion, hypertension, and neonate sex.

Interval: the period between the date of RTIs testing and the date of the last menstrual period of pregnant women
